# Clinical Implementation of Urinary Neutrophil Gelatinase-Associated Lipocalin Testing for Diagnosing Acute Kidney Injury in an Academic Tertiary Care Medical Centre

**DOI:** 10.34067/KID.0000000887

**Published:** 2025-08-13

**Authors:** Michael Strader, Sharjeel Imran, Abdullah Tariq, Candice Fraser, Ellen Saghie, Bernadine C. Louis, Therese Meade, Vladamir Stoyanov, Jean-Maxime Cote, Patrick J. Twomey, Patrick T. Murray

**Affiliations:** 1 School of Medicine, University College Dublin, Dublin, Ireland; 2 St. Vincent's University Hospital, Dublin, Ireland; 3 Division of Nephrology, Department of Medicine, Centre hospitalier de l’Université de Montréal, Montreal, Quebec, Canada

**Keywords:** AKI, biomarkers

## Abstract

**Key Points:**

- Urinary neutrophil gelatinase-associated lipocalin is a sensitive urinary biomarker in the differentiation of intrinsic (intrarenal) AKI from nonintrinsic (prerenal and postrenal) AKI.
- Urinary neutrophil gelatinase-associated lipocalin in addition to fractional excretion of urinary sodium and serum creatinine improves accuracy in differentiating intrinsic from nonintrinsic AKI.

**Background:**

Differentiating functional AKI from structural/intrinsic AKI with tubular injury remains a clinical challenge. Urinary neutrophil gelatinase-associated lipocalin (uNGAL) has shown promise in distinguishing these conditions. This study evaluated the implementation of uNGAL in a heterogeneous medical cohort at an academic tertiary care center in Ireland over a 3-year period.

**Methods:**

A retrospective audit was conducted from 2020 to 2023. Standard clinical data around the time of AKI and uNGAL request were recorded. Blinded case adjudication of the differential diagnosis of AKI cause using the standard clinical information (but not urine neutrophil gelatinase-associated lipocalin results) was performed by two expert nephrologists. Analysis of uNGAL focused on the accuracy in differentiating adjudicated (intrarenal) AKI from nonintrinsic AKI (prerenal and postrenal).

**Results:**

A total of 323 uNGAL tests were performed, with 292 AKI cases adjudicated. Intrinsic AKI cases had significantly higher uNGAL and uNGAL/creatinine ratio (uNGAL/Cr) levels than nonintrinsic cases (*P* < 0.001), including after excluding urinary tract infection cases. uNGAL (area under the receiver operation curve [AUC], 0.71; 95% confidence interval [CI], 0.65 to 0.77) and uNGAL/Cr (AUC, 0.73; 95% CI, 0.67 to 0.79) showed moderate discriminative performance. uNGAL (threshold [Thr] 150 ng/ml) had high sensitivity (0.87) and negative predictive value (0.82). uNGAL/Cr was similar at the 288 ng/mg Thr. Discriminative performance improved for uNGAL and uNGAL/Cr, but not for serum creatinine, fractional excretion of urinary sodium, or serum urea, after excluding urinary tract infection cases. Both uNGAL (adjusted odds ratios, 2.05; 95% CI, 1.59 to 2.71) and uNGAL/Cr (adjusted odds ratios, 2.07; 95% CI, 1.64 to 2.68) were independently associated with intrinsic AKI. Adding these biomarkers to a logistic regression model significantly improved discrimination performance (AUC, 0.79; 95% CI, 0.76 to 0.84; *P* = 0.0116).

**Conclusions:**

The use of uNGAL improved the discriminative accuracy of differential diagnosis of AKI in clinical practice by differentiating intrinsic AKI from nonintrinsic. Specificity was low at the manufacturer's recommended Thr (150 ng/ml), but the sensitivity and negative predictive value were high in all analyses. These findings support the clinical utility of uNGAL at the 150 ng/ml Thr as a “rule-out” test for intrinsic AKI, thereby helping to direct management toward functional (prerenal) or obstructive (postrenal) causes when uNGAL is negative.

## Visual Abstract

**Figure:**
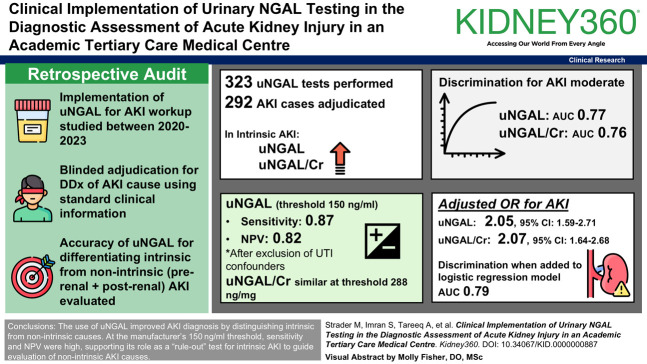

## Introduction

In patients with the syndrome of AKI, it is often difficult to differentiate patients with functional AKI (“prerenal” due to reversible hypoperfusion [*e.g*., hypovolemia, heart failure]) versus intrinsic AKI (“intrarenal” [*e.g*., acute tubular necrosis (ATN)]), particularly for high AKI risk groups like patients with congestive heart failure and liver cirrhosis. The current Kidney Disease Improving Global Outcomes (KDIGO) AKI classification system uses only functional clinical tools (changes in serum creatinine [Cr] and urine output) to diagnose and stage AKI cases. This can lead to difficulty in differentiating phenotypes and any potential subphenotypes.^1–4^ Additional diagnostic tests, such as the fractional excretion of urinary sodium (FENa) and urine microscopy, are often combined with a clinical history and examination to determine the AKI etiology, but all have varying accuracy in differentiating AKI phenotypes.^5–7^

Novel biomarkers have been extensively evaluated and showed promise in providing diagnostic enrichment to differentiate subphenotypes of AKI. Taken together, review of this evidence prompted the 23rd Acute Disease Qualitive Initiative conference to propose modification of the KDIGO AKI diagnostic and staging criteria to include damage biomarkers in combination with standard biomarkers.^8,9^

Kidney biopsies are rarely performed during episodes of AKI; therefore, noninvasive biomarkers such as urinary neutrophil gelatinase-associated lipocalin (uNGAL) have shown promise as early and sensitive diagnostic markers of tubular injury, correlating well with histologic findings.^10,11^ Moreover, uNGAL has demonstrated utility as a diagnostic tool in differentiating functional from intrarenal AKI, identifying AKI subphenotypes, and is cost effective.^12–15^ Recently, the US Food and Drug Association approved uNGAL as a prognostic marker in pediatric intensive care units, and ongoing trials are exploring its utility in adult populations (NCT06652100).^16^ Although uNGAL has been widely studied, there is limited reporting on its implementation as a diagnostic AKI biomarker in routine clinical practice.^1,12,17^

In Ireland, neutrophil gelatinase-associated lipocalin (NGAL) was introduced to a single center for use exclusively on the inpatient renal consult service, and initial results were reported.^18^ Since this initial publication, the broader use outside of the nephrology consult service and the ongoing discriminative performance and accuracy of uNGAL have yet to be reported within this heterogeneous medical population. Therefore, the aim of this audit was to evaluate the discriminative performance and clinical accuracy of uNGAL in a real-world, single-center Irish cohort, compared with standard diagnostic practices.

## Methods

In June 2020, both plasma and urinary NGAL were introduced to the nephrology consult service at St. Vincent's University Hospital (Dublin, Ireland) as clinical tools to assist in AKI workup. The results of the initial audit data, which included 50 medical patients, have previously been reported.^12^

From June 2020 to February 2021, NGAL testing was restricted to the nephrology consult service. After the initial analysis and publication, plasma NGAL was discontinued, while uNGAL was retained as a clinical test due to its superior performance. From March 2021 to August 2022, the use of uNGAL was expanded beyond the nephrology consult service to include all hospital medical specialties. Consequently, a retrospective analysis of uNGAL data from June 2020 to August 2022 was performed at St. Vincent's University Hospital to assess the discriminative performance and diagnostic accuracy of uNGAL since its implementation. The goal of the audit was to further investigate the diagnostic utility of uNGAL to differentiate nonintrinsic AKI, such as either functional/“prerenal” AKI (including hypovolemia, cardiorenal syndrome, and hepatorenal syndrome) or “postrenal”/obstructive AKI, from intrinsic/“intrarenal” AKI with tubular injury in a heterogeneous hospitalized clinical population with AKI. The initial and follow-up audits were approved by the St. Vincent's University Hospital Clinical Audit Committee.

All patients included in the study received inpatient care at St. Vincent's University Hospital. Patient medical record numbers and dates of uNGAL results were retrieved from the hospital's online laboratory results system. Retrospective clinical data—including sex (biologic), age, acute medical history, medical history, and medications at the time of the uNGAL test—were collected from multiple sources, including inpatient and outpatient medical, laboratory, and radiology records.

Cases were classified as having an active infection if there was documentation of antibiotic initiation for suspected infection, the presence of infective symptoms, or a positive microbiologic culture result from any site. Among these, cases with positive blood cultures were categorized as sepsis, while those with positive urine cultures and leukocyturia were categorized as urinary tract infection (UTI).

The results of standard biochemical blood and urinary clinical tests ordered by the clinical team or consult service (Supplemental Table 1) were extracted from the online laboratory results system. Serum Cr and urea levels were recorded from within 24 hours (on the same day) of the AKI event and continued over a 7-day period or until routine blood testing was discontinued by the managing clinical team. Urine was collected according to clinical standards (*e.g*., *via* urinary catheter or “free-catch”) as part of AKI workup. uNGAL testing was ordered by the clinical or nephrology team at any point during the 7-day AKI period. If multiple uNGAL tests were ordered during the same AKI episode, only the result closest to the time of AKI diagnosis was recorded. For patients with multiple AKI episodes, uNGAL results were recorded separately for each event, provided that the patient's serum Cr had returned to baseline between episodes.

### Adjudication

Two expert nephrologists (P.T. Murray and J.M. Cote or V. Stoyanov), who were not involved in patient care at St. Vincent's University Hospital, independently reviewed the acute clinical history, clinical outcomes, and laboratory results for each patient. They were blinded to the uNGAL results and to each other's assessments. Patients were adjudicated as “prerenal,” “intrarenal,” or “postrenal,” “No-AKI,” or “Not Enough Information.” AKI was defined according to KDIGO criteria.^4^

Patients categorized as prerenal were further classified as “Hypovolemia,” “Cardiorenal syndrome,” or “Hepatorenal Syndrome,” depending on the likely cause of the AKI event. Hypovolemia included any decreased perfusion pathologies, such as sepsis-related AKI, if the cases were unlikely to be cardiorenal or hepatorenal. Moreover, if patients were categorized as No-AKI or Not Enough Information, they were further classified as “CKD,” “AKI,” or “Undetermined.”

If consensus was not initially reached, adjudicators independently re-reviewed all available clinical information (excluding uNGAL results and prior opinions) and readjudicated. Patient histories and clinical data were reassessed, incorporating supplemental information such as biopsy results, laboratory data (*e.g*., timing of uNGAL measurement relative to serial serum Cr to provide context around AKI event, but not the uNGAL value), discharge summaries, and radiology findings. In several cases, these data were unavailable at the time of initial adjudication due to delays in hospital processing or delayed availability on the hospital's electronic record system. After readjudication, if consensus was still not achieved the cases were categorized as “undetermined.”

### Assay

uNGAL was measured using the NGAL Test, manufactured by BioPorto Diagnostics (Hellerup, Denmark), measured on the Roche Cobas 8000 c502 automated clinical chemistry analyzer. uNGAL values below the reference linearity ranges were set at the lowest measurable value (25 ng/ml) of the calibration curve. Values above the calibration curve (>3000 ng/ml) were diluted until the measurement was within the linearity. A cutoff value of 150 ng/ml, which was reported as “consistent with AKI” as per the manufacturer, has been previously described.^1^ uNGAL/creatinine ratio (uNGAL/Cr) levels were calculated by dividing uNGAL levels by mg of urinary creatinine.

### Statistical Analysis

Descriptive statistics were reported as means and SD if normally distributed or medians with interquartile ranges (IQRs) if non-normally distributed. Categorical variables were presented as their raw numbers and proportions.

Box-and-whisker plots were generated to compare biomarker results between intrinsic and nonintrinsic AKI categories and stratified by whole population (“ALL”) or population after excluding UTI cases (“No-UTI”). The Shapiro-Wilk test was conducted to assess normality. All biomarkers were approximately non-normally distributed, so nonparametric tests were applied for the analysis. The Mann–Whitney *U* test was performed during intragroup comparisons.

The receiver operating characteristic (ROC) curve, the area under the ROC curve (AUC), confidence intervals (CI), sensitivity (SN), specificity (SP), positive predictive value (PPV), and negative predictive value (NPV) of uNGAL and the urine neutrophil gelatinase-associate liopalin/urinary creatinine ratio were compared with serum Cr, serum urea, and FENa for the diagnosis of intrinsic (intrarenal) versus nonintrinsic (prerenal and postrenal) AKI. Accuracy of uNGAL was explored using predefined thresholds (Thrs) of 150 ng/ml for uNGAL and 288 ng/mg^1^ for uNGAL/Cr. The Youden Index was used to calculate optimal Thrs based on the dataset for uNGAL, uNGAL/Cr, serum Cr, and serum urea.

AUC values were compared with 0.5 (chance) or between the ALL and No-UTI AUCs using the DeLong test.

Adjusted odds ratios were calculated for the association between intrinsic AKI and each biomarker (uNGAL, uNGAL/Cr, and FENa) with covariates used routinely during AKI evaluation (Supplemental Methods).

Multivariable logistic regression models were developed to compare standard biomarkers (serum Cr, urea, FENa) with models including uNGAL (both raw and Cr-corrected), analyzed as continuous and binary variables (based on predefined Thrs). Discriminative performance was evaluated using AUC and diagnostic accuracy metrics, with model comparisons performed using the DeLong test.

Statistical significance was set at *P* < 0.05. All statistics were run on R version 4.4.2 (2024-10-31) using the rstatix,^12^ pROC,^13^ caret,^14^ and tidyverse^15^ packages.

## Results

### Clinical Characteristics of Audit Population

**Figure 1:**
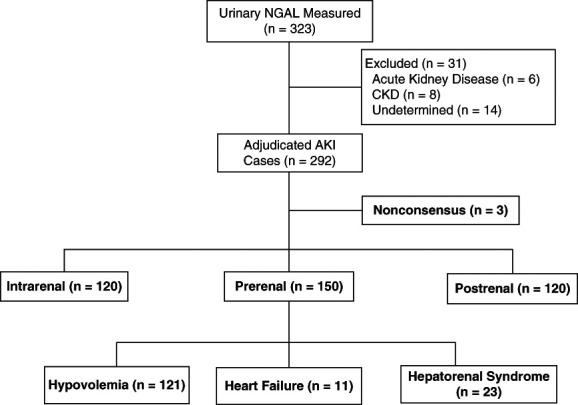
**Diagram depicting the number of uNGAL tests measured during the audit period and cases adjudicated into their AKI cause.** Prerenal cases were further categorized into their most likely cause. Cases categorized as undetermined were due to paucity of case data around the uNGAL measurement. Cases where consensus was not achieved during adjudication were categorized as “nonconsensus” and was further pooled with the “undetermined” category due to paucity of data. uNGAL, urinary neutrophil gelatinase-associated lipocalin.

From the introduction of uNGAL testing until the end of the audit period, 323 uNGAL tests were ordered, with the majority (79.8%) requested within 48 hours of a documented rise in serum Cr. Most tests were performed in cases of AKI (*n*=292; 90.4%), while use in AKI (*n*=6; 1.9%) and CKD (*n*=8; 2.5%) was less common. Eighty cases (27.4%) were readjudicated due to nonconsensus after initial adjudication. Seventeen (5.3%) cases were categorized as undetermined, due to inadequate availability of data required for adjudication or further nonconsensus between adjudicators (Figure 1).

The audit population consisted of 121 women (37.7%) with a mean age of the 66.6 (SD, 16.6) years. The population was heterogeneous with multiple baseline comorbidities increasing the risk for AKI, such as diabetes (*n*=64 [20.5%]), hypertension (*n*=152 [48.4%]), cardiovascular disease (*n*=67 [21.3]), heart failure (*n*=51 [16.3]), liver cirrhosis (59 [19.0%]), and CKD (129 [40.3]). Infection was common at the time of AKI (*n*=200 [62.5%], with UTI occurring in 64 [19.8%] patients (Supplemental Table 2).

Table 1 presents the clinical characteristics of AKI patients, comparing groups adjudicated as intrinsic versus nonintrinsic AKI (prerenal and postrenal). Most patients (58.9%) had nonintrinsic AKI, including prerenal (*n*=151, 51.7%) and postrenal (*n*=22, 7.5%) AKI, while 119 (40.8%) were classified as intrinsic AKI.

**Table 1:** Clinical characteristics of patients stratified by AKI cause

Variable	Nonintrinsic		
	Prerenal (*n*=151)	Postrenal (*n*=22)	Intrinsic (*n*=119)
Sex (female)	63 (42.0)	3 (13.6)	45 (38.1)
Age (mean [SD])	67.0 (16.0)	70.4 (14.2)	65.8 (17.2)
Preadmission serum Cr (μmol/l; median [IQR])	105.0 [72.0–144.0]	140.0 [102.5–170.5]	105.0 [74.0–142.0]
Serum Cr (μmol/L) at KDIGO AKI diagnosis (median [IQR])	229.0 [159.5–300.0]	240.5 [194.8–378.0]	308.5 [233.0–472.0]
ICU admission (*n* [%])	17 (11.6)	1 (4.5)	27 (22.9)
Recent surgery (*n* [%])	34 (22.7)	4 (18.2)	30 (25.2)
Diabetes (*n* [%])	32 (21.8)	4 (18.2)	25 (21.9)
Hypertension (*n* [%])	72 (48.6)	11 (50.0)	55 (47.8)
CVD (*n* [%])	33 (22.8)	5 (22.7)	26 (22.6)
Stroke (*n* [%])	15 (10.1)	2 (9.1)	8 (7.0)
Heart failure (*n* [%])	30 (20.3)	3 (13.6)	16 (13.8)
Liver cirrhosis (*n* [%])	41 (27.9)	0 (0.0)	12 (10.4)
CKD (ml/min per 1.73 m^2^; *n* [%])	62 (41.1)	13 (59.1)	45 (38.1)
**CKD KDIGO stage (*n* [%])**			
3	37 (59.7)	10 (76.9)	28 (60.9)
4	21 (33.9)	3 (23.1)	13 (28.3)
5	4 (6.5)	0 (0.0)	4 (8.9)
**Kidney injury classification at uNGAL**			
AKI	83 (54.9)	8 (36.4)	64 (53.8)
AKI on CKD	68 (45.0)	14 (63.6)	55 (46.2)
**Adjudicated prerenal cause**			
Heart failure	11 (7.3)	—	—
Hepatorenal syndrome	20 (13.2)	—	—
Hypovolemia	120 (79.5)	—	—
**Active infection**	96 (64.4)	11 (50.0)	82 (68.9)
Septicemia	12 (7.9)	2 (9.1)	18 (15.1)
UTI status	36 (23.8)	6 (27.3)	21 (17.6)
Serum WBC at NGAL measurement (×10^9^/ml; mean [SD])	12.8 (8.0)	12.0 (6.5)	14.3 (15.6)
Max serum neutrophil count during AKI (×10^9^/ml; mean [SD])	10.2 (7.2)	10.6 (6.4)	11.1 (7.8)
Maximum serum lymphocyte count during AKI (×10^9^/ml; mean [SD])	1.3 (1.0)	1.9 (2.6)	1.4 (2.0)
Serum CRP (mg/L; median [IQR])	44.0 [12.1–106.7]	78.7 [13.6–156.8]	74.9 [30.3–142.5]
**AKI stage at time of uNGAL (*n* [%])**			
1	46 (30.5)	7 (31.8)	11 (9.2)
2	43 (28.5)	6 (27.3)	18 (15.1)
3	62 (4.1)	9 (40.9)	90 (75.6)
**Max AKI stage (*n* [%])**			
1	42 (27.8)	5 (22.7)	11 (9.2)
2	43 (28.5)	6 (27.3)	21 (17.6)
3	66 (43.7)	11 (50.0)	87 (73.1)
KRT	17 (11.3)	1 (4.5)	42 (35.6)

Normally distributed variables are presented as means and SD, while non-normally distributed variables are presented as medians and interquartile range. Maximum serum neutrophil and lymphocyte count were collected at the time of urinary neutrophil gelatinase-associated lipocalin measurement. Cr, creatinine; CRP, C-reactive protein; CVD, cardiovascular disease; ICU, intensive care unit; IQR, interquartile range; KDIGO, Kidney Disease Improving Global Outcomes; NGAL, neutrophil gelatinase-associated lipocalin; uNGAL, urinary neutrophil gelatinase-associated lipocalin; UTI, urinary tract infection; WBC, white blood cell (×10^9^/ml).

Serum Cr at AKI diagnosis was significantly higher in the intrinsic AKI group (median 308.0 µmol/L [IQR, 233.0–472.0]), compared with prerenal AKI (229.0 µmol/L [IQR, 159.5–300.0]) and postrenal AKI (240 µmol/L [IQR, 194.8–378.0]; *P* < 0.001). However, preadmission serum Cr was not significantly different between groups (*P* = 0.189).

Intensive care unit admission was more frequent in intrinsic AKI patients (22.9%) compared with prerenal (11.6%) and postrenal (4.5%) groups. Similarly, KRT was required more often in intrinsic AKI (35.6%) versus prerenal (11.3%) and postrenal (4.5%) groups.

Patients with intrinsic AKI experienced the highest proportion of moderate-severe (Stage 2/3) AKI (*n*=108, 90.8%), compared with prerenal (*n*=109, 72.2%) and postrenal (*n*=17, 77.2%) groups. Hypertension and cardiovascular disease prevalence was similar across groups, while liver cirrhosis was more common in the prerenal AKI group (*n*=41, 27.9%), compared with the intrinsic AKI (*n*=12, 10.4%) group.

Active infections were prevalent in all groups, affecting 64.4% of prerenal AKI, 50.0% of postrenal AKI, and 68.9% of intrinsic AKI patients. UTIs were common across all groups, with the highest proportion observed in the postrenal AKI group (27.3%), followed by the prerenal (23.8%) and intrinsic AKI groups (17.6%).

Among adjudicated prerenal cases, hypovolemia, which included states with reduced kidney perfusion not due to cardiac or liver dysfunction, was the most common (79.5%) cause of prerenal AKI, followed by hepatorenal syndrome (13.2%) and decompensated heart failure (7.3%).

### Urinary Biomarkers in the Presence or Absence of UTI

**Figure 2:**
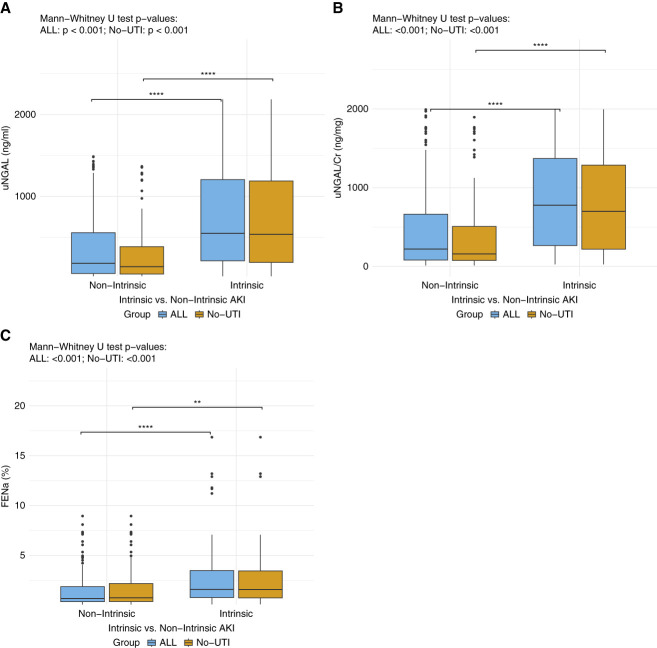
**Adjudicated AKI groups (intrinsic and nonintrinsic) were analyzed before (ALL) and after excluding UTI cases (No-UTI).** Box-and-whisker plots show the 25th–75th IQRs for (A) uNGAL (ng/mL), (B) uNGAL/Cr (ng/mg), and (C) FENa (%). Mann–Whitney *U* tests were used to compare intrinsic versus nonintrinsic groups in both the ALL and No-UTI populations. *P* values are displayed above each plot. Significant results are presented as bars between the compared groups. The asterisk (*) indicate the level of significance: **P < 0.01, ****P < 0.001. uNGAL, uNGAL/Cr, and FENa levels were significantly higher in the intrinsic group compared with the nonintrinsic group even after controlling for UTI status. ALL, whole population; FENa, fractional excretion of urinary sodium; IQR, interquartile range; No-UTI, excluding urinary tract infection; uNGAL/Cr, uNGAL/creatinine ratio; UTI, urinary tract infection.

Figure 2 illustrates the distribution of biomarker levels between intrinsic and nonintrinsic AKI groups, shown separately for the full cohort (ALL) and the subgroup with UTI cases excluded (No-UTI).

Intrinsic AKI patients had significantly higher uNGAL levels (ALL, 1052.4 ng/ml [IQR, 302.4–1314.4]; No-UTI, 930.0 ng/ml [IQR, 296.7–1278.3]) compared with nonintrinsic AKI patients (ALL, 228.2 ng/ml [IQR, 69.7–895.1]; No-UTI, 151.7 ng/ml [IQR, 53.7–420.0]), with significance retained across both comparisons (ALL, *P* < 0.001; No-UTI; *P* < 0.001; Figure 2A).

uNGAL/Cr (Figure 2B) median levels were significantly higher in the intrinsic group (ALL, 1288.7 ng/mg [IQR, 438.2–2317.2]; No-UTI, 1169.0 ng/mg [IQR, 352.3–2093.0]) compared with the nonintrinsic group (ALL, 323.7 ng/mg [IQR, 86.9–1083.7]; No-UTI, 207.2 ng/mg [IQR, 79.1–631.9]). The difference remained significant after excluding UTI cases (ALL, *P* < 0.001) No-UTI, *P* < 0.001).

For FENa (Figure 2C), intrinsic AKI had higher levels (ALL, 1.95% [IQR, 0.97–4.44]; No-UTI, 1.80% [IQR, 0.92–4.38]) compared with nonintrinsic AKI (ALL, 0.82% [IQR, 0.37–2.14]; No-UTI, 0.82% [IQR, 0.37–2.41], *P* < 0.001 for both comparisons).

Intragroup comparisons (ALL versus No-UTI) demonstrated that uNGAL median levels were significantly higher in the nonintrinsic ALL group compared with the nonintrinsic No-UTI group (*P* = 0.044; Supplemental Figure 1). Similarly, uNGAL/Cr levels were significantly higher in the nonintrinsic ALL group (*P* < 0.001) compared with the nonintrinsic No-UTI group. There was no significant difference within intrinsic groups.

### Discriminative Performance and Accuracy of uNGAL and FENa

**Figure 3:**
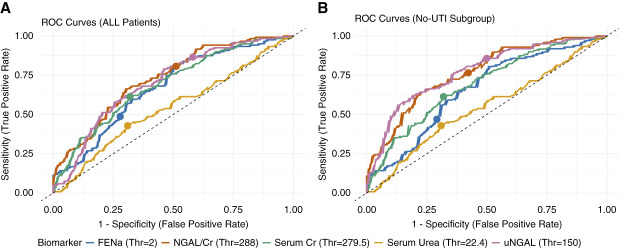
**ROC curves for serum creatinine (green line), serum urea (yellow line), FENa (blue line), uNGAL (pink line), and the uNGAL/Cr (orange line) in discriminating intrinsic from nonintrinsic AKI.** (A) ROC curves for the entire study population; (B) Curves after excluding patients with UTIs. Points on each curve indicate the predefined diagnostic Thr, or Youden Thr for serum creatinine and urea, for the corresponding biomarker. Cr, creatinine; ROC, receiver operating characteristics; Thr, threshold.

The ROC curves for each biomarker are presented in Figure 3, illustrating their ability to differentiate intrinsic from nonintrinsic AKI. Table 2 summarizes the corresponding AUCs and diagnostic accuracy metrics for each biomarker. Serum Cr demonstrated poor discriminative performance (AUC, 0.69; 95% CI 0.63 to 0.75; *P* < 0.001) for distinguishing intrinsic from nonintrinsic AKI, and at a Youden Thr of 279.50 had a SN of 0.61, SP of 0.68, PPV of 0.57, and NPV of 0.72. Moreover, serum urea showed worse discrimination (AUC, 0.54; 95% CI, 0.47 to 0.61]; *P* = 0.22) and accuracy with a SN of 0.43, SP of 0.69, PPV of 0.49, and NPV of 0.64 at a Youden Thr of 22.45 mmol/L.

**Table 2:** Discriminative ability and accuracy of neutrophil gelatinase-associated lipocalin and fractional excretion of urinary sodium for differentiating intrinsic from nonintrinsic AKI

Group (All *N*=292; No-UTI *N*=229)	AUC (95% CI)	SN	SP	PPV	NPV	Calculated Youden Thr
**Serum Cr (μmol/L)**						
ALL	0.69 (0.63 to 0.75)	0.61	0.68	0.57	0.72	279.50 mmol/L
No-UTI	0.69 (0.62 to 0.76)	0.68	0.61	0.57	0.72	259.50 mmol/L
**Serum urea**						
ALL	0.54 (0.47 to 0.61)	0.43	0.69	0.49	0.64	22.5 mmol/L
No-UTI	0.54 (0.45 to 0.61)	0.43	0.67	0.49	0.61	22.5 mmol/L
**uNGAL (150 ng/ml)**						
ALL	0.71 (0.65 to 0.76)	0.87	0.42	0.51	0.82	—
No-UTI	0.77 (0.71 to 0.83)	0.86	0.50	0.56	0.82	—
**uNGAL (ng/ml)**						
ALL	0.71 (0.64 to 0.77)	0.62	0.71	0.64	0.70	690 ng/ml
No-UTI	0.77 (0.71 to 0.84)	0.63	0.82	0.76	0.72	590 ng/ml
**Cr corrected uNGAL (ng/mg)**						
ALL	0.73 (0.68 to 0.79)	0.67	0.71	0.65	0.73	750 ng/mg
No-UTI	0.77 (0.71 to 0.83)	0.65	0.80	0.74	0.72	680 ng/mg
**Cr corrected uNGAL (288 ng/mg)**						
ALL	0.73 (0.67 to 0.79)	0.81	0.49	0.52	0.79	
No-UTI	0.76 (0.70 to 0.83)	0.77	0.58	0.58	0.77	
**FENa (2%)**						
ALL	0.67 (0.61 to 0.73)	0.49	0.72	0.55	0.67	—
No-UTI	0.65 (0.58 to 0.73)	0.47	0.71	0.55	0.64	—

Calculated metrics before (whole population) and after excluding urinary tract infection cases. Serum creatinine and urea measured on the day of AKI diagnosis are provided as reference. AUC for all biomarkers, whole population, and excluding urinary tract infections, compared with 0.5 (random) were statistically significant (*P* < 0.001), except serum urea (*P* = 0.22). There was no statistical difference between DeLong *P* values comparing AUC between the whole population and excluding urinary tract infection for each biomarker and the thresholds. ALL, whole population; AUC, area under the receiver operation curve; CI, confidence interval; Cr, creatinine; FENa, fractional excretion of urinary sodium; SN, sensitivity; SP, specificity; PPV, positive predictive value; No-UTI, excluding urinary tract infection; NPV, negative predictive value; Thr, threshold; uNGAL, urinary neutrophil gelatinase-associated lipocalin.

uNGAL demonstrated higher discriminative performance (AUC, 0.71; 95% CI, 0.65 to 0.76; *P* < 0.001) and accuracy compared with the standard biomarkers in differentiating intrinsic from nonintrinsic AKI. At the manufacturer-recommended Thr of 150 ng/ml, uNGAL achieved a SN of 0.87, SP of 0.42, PPV of 0.51, and NPV of 0.82.

uNGAL/Cr demonstrated good discriminative performance (AUC, 0.73; 95% CI, 0.67 to 0.79; *P* < 0.001) and diagnostic accuracy at a Thr of 288 ng/mg, with a SN of 0.81, SP of 0.49, PPV of 0.52, and NPV of 0.79, respectively. The Youden Thr of 750 ng/ml SP (0.71) and PPV (0.64) were higher, while SN (0.67) and NPV (0.70) decreased.

By contrast, FENa had poor discriminative performance (AUC, 0.67; 95% CI, 0.61 to 0.73; *P* < 0.001), and at the 2%, Thr had lower SN (0.49) and NPV (0.67), but higher SP (0.72) and PPV (0.55).

Excluding UTI cases had minimal effect on the discriminative performance of serum Cr (AUC, 0.69; 95% CI, 0.62 to 0.76), serum urea (AUC, 0.53; 95% CI, 0.45 to 0.61), and FENa (AUC, 0.65; 95% CI, 0.58 to 0.73). By contrast, the performance of both uncorrected and Cr-corrected uNGAL improved, although these differences did not reach statistical significance (*P* = 0.1267 for both; Figure 3B and Table 2).

The AUC for uNGAL increased to 0.77 (95% CI, 0.71 to 0.83) after excluding UTI cases. The exclusion of UTI cases had little effect on SN (0.86) and NPV (0.82) at the 150 ng/ml Thr. The Youden-derived Thr (590 ng/ml) yielded high SP (0.82) and PPV (0.76), while SN (0.63) and NPV (0.72) were comparable with values observed when UTI cases were included.

For uNGAL/Cr, the AUC increased to 0.76 (95% CI, 0.66 to 0.80) after excluding UTI cases. The exclusion of UTI cases had little effect on diagnostic accuracy at the 288 ng/mg Thr, where SN remained moderate (0.77), and SP remained poor (0.58). NPV and PPV remained similar at 0.77 and 0.58, respectively. The Youden-derived Thr (680 ng/mg) yielded higher SP (0.80) and PPV (0.74), while SN (0.65) and NPV (0.72) remained comparable with values including UTI cases.

### Intrarenal AKI Diagnosis Prediction Model

aOR demonstrated a significant association between higher biomarker levels and the diagnosis of intrinsic AKI. Both uNGAL (aOR, 2.05; 95% CI, 1.59 to 2.71; *P* < 0.001) and uNGAL/Cr (aOR, 2.07; 95% CI, 1.64 to 2.68; *P* < 0.001) were strongly associated with intrinsic AKI. FENa also showed a significant association with intrinsic AKI (aOR, 1.53; 95% CI, 1.20–1.97; *P* = 0.0008).

A multivariate logistic regression model using just serum Cr, serum urea (both measured on the day of AKI diagnosis), and FENa, which are standard variables used in most clinical centers for AKI, yielded an AUC of 0.72 (95% CI, 0.66 to 0.78), with a SN of 0.62, SP of 0.79, PPV of 0.68, and NPV of 0.75. The addition of uNGAL (Thr 150 ng/ml) and uNGAL/Cr (Thr 288 ng/mg) improved the model's discriminative performance (AUC, 0.76; 95% CI, 0.71 to 0.82), with a high SN (0.86) and NPV (0.85), but lower SP (0.53) and PPV (0.56). However, this did not reach statistical significance compared with the standard biomarker model (*P* = 0.0785).

If uNGAL and uNGAL/Cr are added to the model as continuous variables, discriminative performance significantly improves (AUC, 0.79; 95% CI, 0.76 to 0.84; *P* = 0.0116) compared with the standard biomarker model. Moreover, the NPV (0.80) and SN (0.76) improved, but SP (0.69) and PPV (0.63) decreased.

## Discussion

In a heterogeneous AKI population, distinguishing between nonintrinsic (prerenal and postrenal) and intrinsic AKI is critical for effective patient management. A key limitation of the functional biomarker serum Cr is its inability to distinguish between AKI phenotypes, particularly intrinsic versus nonintrinsic causes. As a result, novel biomarkers, such as uNGAL, have been proposed to augment the KDIGO criteria and add diagnostic granularity to the heterogeneous syndrome of AKI.^2,9^

In this audit, we demonstrated that uNGAL and uNGAL/Cr, when measured around the time of acute Cr-defined AKI, improved discriminative performance and diagnostic accuracy in differentiating intrinsic from nonintrinsic AKI when used alongside standard clinical tools. The accuracy of uNGAL in this audit was consistent with previously reported clinical implementation studies.^1,12^ At the Thrs identified in this cohort, there is a clear trade-off: lower Thrs improve SN but reduce SP. The cutoffs derived from the Youden index were higher than previously documented values but maintained an increase in SP at higher Thrs at the expense of SN.^1^

Recently, uNGAL levels were assessed across the age spectrum in self-reported “healthy” participants, with most adult values falling below 150 ng/ml.^19^ However, in clinical practice, patients often present with multiple comorbidities that can influence uNGAL concentrations, potentially necessitating higher diagnostic Thrs. Nonetheless, at the lower manufacturer's Thr of 150 ng/ml, the high SN and NPV of uNGAL support its use as a “rule-out” test for intrinsic renal injury—a strategy that has also been applied in pediatric populations—and may aid clinical decision-making, such as determining the appropriateness of initiating terlipressin in cases of suspected hepatorenal syndrome.^19–21^ Future research is needed to establish consensus Thrs by evaluating uNGAL levels in both healthy adults and stable populations with chronic diseases such as CKD.

An important component of uNGAL interpretation is the concomitant UTIs, at the time of uNGAL measurement, which was a significant confounder in this audit. These findings are consistent with previous studies demonstrating that inflammation of the urothelium, such as UTIs, can elevate uNGAL levels and reduce accuracy,^1,12,22,23^ Moreover, there is debate among the literature about the use of Cr-corrected biomarker values versus uncorrected values.^24^ In this cohort, there is value in having both, which was evident by increased discriminative performance in the multivariate logistic regression model when used together.

Clinically, the use of FENa, although not as sensitive at the clinical Thr of 2%, had good SP but had limitations related to multiple confounders.^6,7,25^ Despite these limitations, uNGAL—alongside traditional markers, such as FENa—enhances SN and improves the differential diagnostic assessment of complex AKI phenotypes. In support of this, a previous study found that when nephrologists were asked whether uNGAL assisted in the differential diagnosis or altered clinical management, the majority responded affirmatively.^1^ This refinement helps focus diagnostic workup and management strategies on reversing kidney hypoperfusion (hypovolemia, hepatorenal, cardiorenal) or urinary tract obstruction when intrinsic AKI is unlikely based on uNGAL testing and other information.

For example, in patients with liver cirrhosis and AKI, the use of uNGAL has shown great utility in differentiating the difficult clinical diagnosis of functional hepatorenal syndrome (which does not reverse with fluid therapy alone) from ATN. The correct subphenotyping can guide clinical management using fluid resuscitation (albumin) alone for hypovolemia, or addition of vasopressor therapy (*e.g*., terlipressin) for fluid-refractory hepatorenal syndrome, versus supportive care and additional diagnostic testing for intrinsic AKI. The reassurance of a negative NGAL in this clinical context supports the nonintrinsic AKI component and can guide therapeutic choice.^26–28^ Interestingly, the Thrs for uncorrected uNGAL in the cirrhosis population tend to be higher at 220 ng/ml, which was highlighted in a recent meta-analysis.^14^ Similarly, in patients with acute heart failure and AKI, the development of AKI during escalated diuretic therapy can sometimes prevent effective decongestion because of concerns of superimposed ATN. In this scenario, using a sensitive damage biomarker (*e.g*., a negative uNGAL test) with high NPV can provide reassurance that continued aggressive diuresis remains appropriate and will improve outcomes.^18,29^

This audit has several limitations, including a limited sample size and single-center design, as well as changing uNGAL use patterns across the audit period. The timing of uNGAL measurement was not standardized—some tests were performed on the day of AKI diagnosis, while others occurred days later—which may have introduced variability in biomarker interpretation. However, this reflects current routine in-hospital practices. In addition, the exclusion of UTI cases to generate the No-UTI group and the subsequent reanalysis of discriminative performance and accuracy may have introduced some bias; however, this was likely minimal, as evidenced by the negligible effect on the performance of standard biomarkers.

The inclusion of postrenal obstructive nephropathy, which were largely due to prostatic hypertrophy in males or malignancy in both men and women, in the nonintrinsic group could have influenced the uNGAL levels within the group as they have shown to be elevated in kidney obstructive pathologies.^30,31^

Sample size limitations were unavoidable at this early stage of uNGAL implementation in clinical practice, which remains relatively uncommon worldwide; broader clinical use and validation are encouraged. Diagnostic adjudication and subgroup analysis by infection/inflammation status were conducted to adjust for clinical confounders. In some cases, differences in interpretation between adjudicators, which could have been due to delays in the availability of clinical information, necessitated readjudication, which may have influenced final categorization. However, these limitations reflect the real-world variability in clinician interpretation, clinical data availability, and the complexity of diagnostic decision making in routine practice. Finally, systemic inflammation, such as sepsis, in nonintrinsic AKI may have contributed to elevated uNGAL levels, potentially affecting overall test accuracy.^32–34^

**Figure 4:**
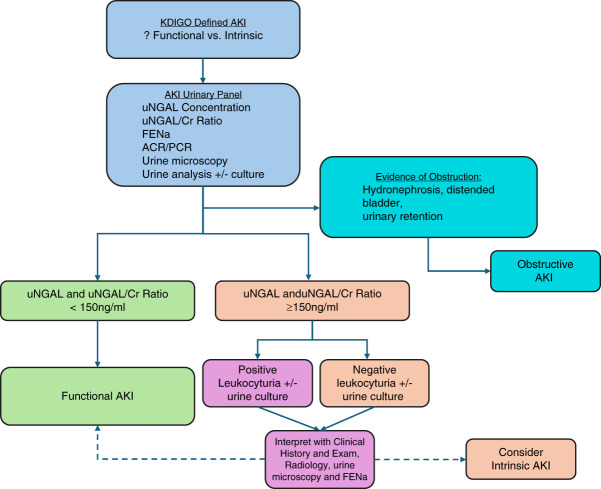
**Example flow diagram for the use of uNGAL in AKI.** Urine analysis includes presence or absence of leukocytes. The presence of leukocyturia can raise uNGAL levels (creatinine corrected and/or uncorrected) and therefore clinical interpretation with history, examination, radiologic findings, and FENa should be used to distinguish functional from intrinsic. UNGAL/Cr can be applied similarly. ACR, albumin/creatinine ratio; KDIGO, Kidney Disease Improving Global Outcomes; PCR, protein/creatinine ratio.

Despite these limitations, the diagnostic performance of uNGAL remained robust, supporting its role in distinguishing intrinsic from nonintrinsic AKI. Notably, when measured at the time of Cr-defined AKI, uNGAL at the manufacturer's 150 ng/ml Thr serves as a sensitive tool to help rule out intrinsic injury in clinical practice. However, it is not a perfect test, as uNGAL levels can be affected by urinary tract inflammation. Therefore, obtaining a urine analysis to measure leukocytes, nitrites, and culture, along with concurrent measurement of FENa, is recommended to enhance clinical interpretation and diagnostic accuracy. Figure 4 presents a proposed flow diagram for the clinical use of uNGAL in KDIGO-defined AKI to aid in differentiating intrinsic from functional AKI. When uNGAL—whether Cr-corrected or uncorrected—is below the diagnostic Thr, a diagnosis of functional AKI is supported. Conversely, if uNGAL levels exceed the Thr, interpretation of the urine analysis for the presence of leukocyturia is necessary to rule out confounders before making a diagnosis of intrinsic AKI.

As biomarkers become further integrated into clinical decision making, additional research is needed to refine uNGAL Thrs, which may vary based on underlying disease pathology and specific clinical differential diagnoses, such as hepatorenal syndrome versus ATN, or hypovolemic AKI versus sepsis-related ATN. Moreover, a reactive, automated panel-based approach incorporating multiple biomarkers at the time of Cr-defined AKI diagnosis may further enhance diagnostic accuracy and better guide timely clinical management. The integration of novel biomarkers into standard clinical pathways marks an important step toward precision diagnostics and the future potential for biomarker-guided therapies.

## Supplementary Material

SUPPLEMENTARY MATERIAL

## Data Availability

Original data generated for the study will be made available on reasonable request to the corresponding author. Health Care Data. Anonymized data are available on reasonable request to the corresponding author.

## Declarative Statements

This study is exempt from the Institutional Review Board or Ethics Committee approval, and I have provided the reason for exemption. Clinical Audit.

## Supplemental Material

Supplemental Table 1. Biochemical data collected from hospital records.

Supplemental Table 2. Clinical characteristics of population.

Supplemental Methods. Covariates for adjusted models.

Supplemental Figure 1. Comparison of urinary biomarker levels between adjudicated prerenal, intrarenal, and postrenal groups with and without UTI.

Supplemental STROBE Ckecklist

## References

[1] Côté J-M Authier R Ethier I, . Clinical implementation of NGAL testing to improve diagnostic assessment of AKI episodes in a canadian center. Can J Kidney Health Dis. 2022;9:20543581221118991. doi:10.1177/20543581221118991 36004277 PMC9393659

[2] Ostermann M Lumlertgul N Jeong R See E Joannidis M James M. Acute kidney injury. Lancet. 2025;405(10474):241–256. doi:10.1016/S0140-6736(24)02385-7 39826969

[3] Rodrigues CE Endre ZH. Definitions, phenotypes, and subphenotypes in acute kidney injury-moving towards precision medicine. Nephrology (Carlton). 2023;28(2):83–96. doi:10.1111/nep.14132 36370326 PMC10100386

[4] Group KDIGOKAKIW. KDIGO clinical practice guideline for acute kidney injury. Kidney Int Suppl. 2012;2(1):1–138. doi:10.1038/kisup.2012.1

[5] Bagshaw SM Haase M Haase-Fielitz A Bennett M Devarajan P Bellomo R. A prospective evaluation of urine microscopy in septic and non-septic acute kidney injury. Nephrol Dial Transplant. 2012;27(2):582–588. doi:10.1093/ndt/gfr331 21669886

[6] Varghese V Rivera MS Alalwan AA Alghamdi AM Gonzalez ME Velez JCQ. Diagnostic utility of serial microscopic examination of the urinary sediment in acute kidney injury. Kidney360. 2021;2(2):182–191. doi:10.34067/KID.0004022020 35373012 PMC8741003

[7] Varghese V Rivera MS Alalwan A, . Concomitant identification of muddy brown granular casts and low fractional excretion of urinary sodium in AKI. Kidney360. 2022;3(4):627–635. doi:10.34067/KID.0005692021 35721603 PMC9136894

[8] Murray PT Mehta RL Shaw A, . Potential use of biomarkers in acute kidney injury: report and summary of recommendations from the 10th acute dialysis quality initiative consensus conference. Kidney Int. 2014;85(3):513–521. doi:10.1038/ki.2013.374 24107851 PMC4198530

[9] Ostermann M Zarbock A Goldstein S, . Recommendations on acute kidney injury biomarkers from the acute disease quality initiative consensus conference: a consensus statement. JAMA Netw Open. 2020;3(10):e2019209. doi:10.1001/jamanetworkopen.2020.19209 33021646

[10] Moledina DG Hall IE Thiessen-Philbrook H, . Performance of serum creatinine and kidney injury biomarkers for diagnosing histologic acute tubular injury. Am J Kidney Dis. 2017;70(6):807–816. doi:10.1053/j.ajkd.2017.06.031 28844586 PMC5701867

[11] Endre ZH. Kidney biopsy in acute kidney injury: increase insight or preserve the status quo? Kidney Int. 2025;107(3):397–400. doi:10.1016/j.kint.2024.09.022 39984253

[12] Côté JM Lyons L Twomey PJ, . Clinical implementation and initial experience of neutrophil gelatinase-associated lipocalin testing for the diagnostic and prognostic assessment of acute kidney injury events in hospitalized patients. Nephron. 2022;146(3):306–314. doi:10.1159/000518611 34515166

[13] Szumilas D Owczarek AJ Brzozowska A Niemir ZI Olszanecka-Glinianowicz M Chudek J. The value of urinary NGAL, KIM-1, and IL-18 measurements in the early detection of kidney injury in oncologic patients treated with cisplatin-based chemotherapy. Int J Mol Sci. 2024;25(2):1074. doi:10.3390/ijms25021074 38256147 PMC10816507

[14] Puthumana J Lugon NC Xu Y, . Systematic review and meta-analysis of urine neutrophil gelatinase-associated lipocalin for acute kidney injury in cirrhosis. Kidney Int Rep. 2024;9(7):2278–2281. doi:10.1016/j.ekir.2024.04.050 39081737 PMC11284356

[15] Parikh A Rizzo JA Canetta P, . Does NGAL reduce costs? A cost analysis of urine NGAL (uNGAL) & serum creatinine (sCr) for acute kidney injury (AKI) diagnosis. PLoS One. 2017;12(5):e0178091. doi:10.1371/journal.pone.0178091 28542336 PMC5438176

[16] Inc BD. ProNephro AKI™ (NGAL): 510(k) Substantial Equivalence Determination Decision Summary; 2024. Accessed November 11, 2024. https://www.fda.gov/medical-devices/recently-approved-devices/k232761-pronephro-aki-ngal

[17] Goldstein SL Akcan-Arikan A Afonso N, . Derivation and validation of an optimal neutrophil gelatinase-associated lipocalin cutoff to predict stage 2/3 acute kidney injury (AKI) in critically ill children. Kidney Int Rep. 2024;9(8):2443–2452. doi:10.1016/j.ekir.2024.05.010 39156146 PMC11328761

[18] Duff S Wettersten N Horiuchi Y, . Absence of kidney tubular injury in patients with acute heart failure with acute kidney injury. Circ Heart Fail. 2024;17(11):e011751. doi:10.1161/CIRCHEARTFAILURE.123.011751 39421939 PMC11573103

[19] Baker TM Bird CA Broyles DL Klause U. Determination of urinary neutrophil gelatinase-associated lipocalin (uNGAL) reference intervals in healthy adult and pediatric individuals using a particle-enhanced turbidimetric immunoassay. Diagnostics (Basel). 2025;15(1):95. doi:10.3390/diagnostics15010095 39795623 PMC11720492

[20] Stanski N Menon S Goldstein SL Basu RK. Integration of urinary neutrophil gelatinase-associated lipocalin with serum creatinine delineates acute kidney injury phenotypes in critically ill children. J Crit Care. 2019;53:1–7. doi:10.1016/j.jcrc.2019.05.017 31174170

[21] Gambino C Piano S Stenico M, . Diagnostic and prognostic performance of urinary neutrophil gelatinase-associated lipocalin in patients with cirrhosis and acute kidney injury. Hepatology. 2023;77(5):1630–1638. doi:10.1002/hep.32799 36125403 PMC10113003

[22] Decavele A-SC Dhondt L De Buyzere ML Delanghe JR. Increased urinary neutrophil gelatinase associated lipocalin in urinary tract infections and leukocyturia. Clin Chem Lab Med. 2011;49(6):999–1003. doi:10.1515/CCLM.2011.156 21391867

[23] Cullen MR Murray PT Fitzgibbon MC. Establishment of a reference interval for urinary neutrophil gelatinase-associated lipocalin. Ann Clin Biochem. 2012;49(Pt 2):190–193. doi:10.1258/acb.2011.011105 22323662

[24] Tang KW Toh QC Teo BW. Normalisation of urinary biomarkers to creatinine for clinical practice and research--when and why. Singapore Med J. 2015;56(1):7–10. doi:10.11622/smedj.2015003 25640093 PMC4325562

[25] Nadim MK Kellum JA Forni L, . Acute kidney injury in patients with cirrhosis: acute disease quality initiative (ADQI) and International Club of Ascites (ICA) joint multidisciplinary consensus meeting. J Hepatol. 2024;81(1):163–183. doi:10.1016/j.jhep.2024.03.031 38527522 PMC11193657

[26] Allegretti AS Parada XV Endres P, . Urinary NGAL as a diagnostic and prognostic marker for acute kidney injury in cirrhosis: a prospective Study. Clin Transl Gastroenterol. 2021;12(5):e00359. doi:10.14309/ctg.0000000000000359 33979307 PMC8116001

[27] Allegretti AS Israelsen M Krag A, . Terlipressin versus placebo or no intervention for people with cirrhosis and hepatorenal syndrome. Cochrane Database Syst Rev. 2017;6(6):Cd005162. doi:10.1002/14651858.CD005162.pub4 29943803 PMC6481608

[28] Agrawal N Louis-Jean S Ladiwala Z, . Reliability of neutrophil gelatinase-associated lipocalin in detecting acute tubular necrosis in decompensated cirrhosis: systematic review and meta-analysis. World J Hepatol. 2024;16(11):1331–1338. doi:10.4254/wjh.v16.i11.1331 39606167 PMC11586751

[29] Horiuchi Y Wettersten N van Veldhuisen DJ, . Decongestion, kidney injury and prognosis in patients with acute heart failure. Int J Cardiol. 2022;354:29–37. doi:10.1016/j.ijcard.2022.02.026 35202737

[30] Sise ME Forster C Singer E, . Urine neutrophil gelatinase-associated lipocalin identifies unilateral and bilateral urinary tract obstruction. Nephrol Dial Transplant. 2011;26(12):4132–4135. doi:10.1093/ndt/gfr569. 22049182 PMC3254163

[31] Brewin A Sriprasad S Somani B. The use of neutrophil gelatinase-associated lipocalin (NGAL) as a diagnostic and prognostic biomarker in urinary tract obstruction: a systematic review. Curr Urol Rep. 2022;23(8):155–163. doi:10.1007/s11934-022-01098-6 35678987

[32] Vanmassenhove J Glorieux G Lameire N, . Influence of severity of illness on neutrophil gelatinase-associated lipocalin performance as a marker of acute kidney injury: a prospective cohort study of patients with sepsis. BMC Nephrol. 2015;16(1):18. doi:10.1186/s12882-015-0003-y 25868473 PMC4352556

[33] Dellepiane S Marengo M Cantaluppi V. Detrimental cross-talk between sepsis and acute kidney injury: new pathogenic mechanisms, early biomarkers and targeted therapies. Crit Care. 2016;20:61. doi:10.1186/s13054-016-1219-3 26976392 PMC4792098

[34] Lentini P de Cal M Clementi A D'Angelo A Ronco C. Sepsis and AKI in ICU patients: the role of plasma biomarkers. Crit Care Res Pract. 2012;2012(1):856401. doi:10.1155/2012/856401 22400110 PMC3286882

