# Supplementary material for: Clinical Implementation of Urinary Neutrophil Gelatinase-Associated Lipocalin Testing for Diagnosing Acute Kidney Injury in an Academic Tertiary Care Medical Centre
Source: Kidney360. 2025 Aug 13;6(12):2119–29. doi: 10.34067/KID.0000000887 (PMC12708381; doi:10.34067/KID.0000000887)
Supplement: SUPPLEMENTARY MATERIAL [file kidney360-6-2119-s001.pdf]

ASN American Society of Nephrology | JASN Journal of the American Society of Nephrology | KIDNEY360 Accessing Our World From Every Angle | CJASN Clinical Journal of the American Society of Nephrology

ASN Journal Disclosure Form

As per ASN journal policy, I have disclosed any financial relationships or commitments I have held in the past 36 months as included below. I have listed my Current Employer below to indicate there is a relationship requiring disclosure. If no relationship exists, my Current Employer is not listed.

J. Cote reports the following:
Employer: CHUM; and Other Interests or Relationships: Member - Canadian Society of Nephrology; Nephrology Consultant - CHUM (Montreal).

I understand that the information above will be published within the journal article, if accepted, and that failure to comply and/or to accurately and completely report the potential financial conflicts of interest could lead to the following: 1) Prior to publication, article rejection, or 2) Post-publication, sanctions ranging from, but not limited to, issuing a correction, reporting the inaccurate information to the authors' institution, banning authors from submitting work to ASN journals for varying lengths of time, and/or retraction of the published work.

Name: Jean Maxime Cote
Manuscript ID: K360-2025-000331
Manuscript Title: Clinical Implementation of uNGAL Testing for Diagnosing Acute Kidney Injury in an Academic Tertiary Care Medical Centre
Date of Completion: March 13, 2025
Disclosure Updated Date: March 13, 2025

ASN American Society of Nephrology | JASN Journal of the American Society of Nephrology | KIDNEY360 Accessing Our World From Every Angle | CJASN Clinical Journal of the American Society of Nephrology

ASN Journal Disclosure Form

As per ASN journal policy, I have disclosed any financial relationships or commitments I have held in the past 36 months as included below. I have listed my Current Employer below to indicate there is a relationship requiring disclosure. If no relationship exists, my Current Employer is not listed.

C. Fraser has nothing to disclose.

I understand that the information above will be published within the journal article, if accepted, and that failure to comply and/or to accurately and completely report the potential financial conflicts of interest could lead to the following: 1) Prior to publication, article rejection, or 2) Post-publication, sanctions ranging from, but not limited to, issuing a correction, reporting the inaccurate information to the authors' institution, banning authors from submitting work to ASN journals for varying lengths of time, and/or retraction of the published work.

Name: Candice Neezeth Fraser
Manuscript ID: K360-2025-000331
Manuscript Title: Clinical Implementation of uNGAL Testing for Diagnosing Acute Kidney Injury in an Academic Tertiary Care Medical Centre
Date of Completion: May 10, 2025
Disclosure Updated Date: May 10, 2025

ASN American Society of Nephrology | JASN Journal of the American Society of Nephrology | KIDNEY360 Accessing Our World From Every Angle | CJASN Clinical Journal of the American Society of Nephrology

ASN Journal Disclosure Form

As per ASN journal policy, I have disclosed any financial relationships or commitments I have held in the past 36 months as included below. I have listed my Current Employer below to indicate there is a relationship requiring disclosure. If no relationship exists, my Current Employer is not listed.

S. Imran has nothing to disclose.

I understand that the information above will be published within the journal article, if accepted, and that failure to comply and/or to accurately and completely report the potential financial conflicts of interest could lead to the following: 1) Prior to publication, article rejection, or 2) Post-publication, sanctions ranging from, but not limited to, issuing a correction, reporting the inaccurate information to the authors' institution, banning authors from submitting work to ASN journals for varying lengths of time, and/or retraction of the published work.

Name: Sharjeel Imran
Manuscript ID: K360-2025-000331
Manuscript Title: Clinical Implementation of uNGAL Testing for Diagnosing Acute Kidney Injury in an Academic Tertiary Care Medical Centre
Date of Completion: March 18, 2025
Disclosure Updated Date: March 18, 2025

ASN American Society of Nephrology JASN Journal of the American Society of Nephrology KIDNEY360 Accessing Our World From Every Angle CJASN Clinical Journal of the American Society of Nephrology

ASN Journal Disclosure Form

As per ASN journal policy, I have disclosed any financial relationships or commitments I have held in the past 36 months as included below. I have listed my Current Employer below to indicate there is a relationship requiring disclosure. If no relationship exists, my Current Employer is not listed.

B. Louis has nothing to disclose.

I understand that the information above will be published within the journal article, if accepted, and that failure to comply and/or to accurately and completely report the potential financial conflicts of interest could lead to the following: 1) Prior to publication, article rejection, or 2) Post-publication, sanctions ranging from, but not limited to, issuing a correction, reporting the inaccurate information to the authors' institution, banning authors from submitting work to ASN journals for varying lengths of time, and/or retraction of the published work.

Name: Bernadine Carolina Louis
Manuscript ID: K360-2025-000331
Manuscript Title: Clinical Implementation of uNGAL Testing for Diagnosing Acute Kidney Injury in an Academic Tertiary Care Medical Centre
Date of Completion: May 10, 2025
Disclosure Updated Date: May 10, 2025

ASN American Society of Nephrology | JASN Journal of the American Society of Nephrology | KIDNEY360 Accessing Our World From Every Angle | CJASN Clinical Journal of the American Society of Nephrology

ASN Journal Disclosure Form

As per ASN journal policy, I have disclosed any financial relationships or commitments I have held in the past 36 months as included below. I have listed my Current Employer below to indicate there is a relationship requiring disclosure. If no relationship exists, my Current Employer is not listed.

T. Meade reports the following:
Employer: Mater Misercordiae University Hospital; University College Dublin

I understand that the information above will be published within the journal article, if accepted, and that failure to comply and/or to accurately and completely report the potential financial conflicts of interest could lead to the following: 1) Prior to publication, article rejection, or 2) Post-publication, sanctions ranging from, but not limited to, issuing a correction, reporting the inaccurate information to the authors' institution, banning authors from submitting work to ASN journals for varying lengths of time, and/or retraction of the published work.

Name: Tess Rose Meade
Manuscript ID: K360-2025-000331
Manuscript Title: Clinical Implementation of uNGAL Testing for Diagnosing Acute Kidney Injury in an Academic Tertiary Care Medical Centre
Date of Completion: March 13, 2025
Disclosure Updated Date: March 13, 2025

ASN American Society of Nephrology | JASN Journal of the American Society of Nephrology | KIDNEY360 Accessing Our World From Every Angle | CJASN Clinical Journal of the American Society of Nephrology

ASN Journal Disclosure Form

As per ASN journal policy, I have disclosed any financial relationships or commitments I have held in the past 36 months as included below. I have listed my Current Employer below to indicate there is a relationship requiring disclosure. If no relationship exists, my Current Employer is not listed.

P. Murray reports the following:
Employer: Merck (Spouse); Consultancy: Renibus Therapeutics, Novartis, Alexion, Calcimedica, Bioporto Diagnostics, Pfizer; Ownership Interest: Merck (spouse); CalciMedica (Patrick Murray); Research Funding: Abbott Laboratories (grants to employer/institution); and Advisory or Leadership Role: Accreditation Commission on Colleges of Medicine; paid role.; Oversight Committee, Biomarker Data Repository (BmDR); unpaid role (pro bono).

I understand that the information above will be published within the journal article, if accepted, and that failure to comply and/or to accurately and completely report the potential financial conflicts of interest could lead to the following: 1) Prior to publication, article rejection, or 2) Post-publication, sanctions ranging from, but not limited to, issuing a correction, reporting the inaccurate information to the authors' institution, banning authors from submitting work to ASN journals for varying lengths of time, and/or retraction of the published work.

Name: Patrick T. Murray
Manuscript ID: K360-2025-000331
Manuscript Title: Clinical Implementation of uNGAL Testing for Diagnosing Acute Kidney Injury in an Academic Tertiary Care Medical Centre
Date of Completion: March 13, 2025
Disclosure Updated Date: March 12, 2025

ASN
American Society of Nephrology
JASN
Journal of the American Society of Nephrology
KIDNEY360
Accessing Our World From Every Angle
CJASN
Clinical Journal of the American Society of Nephrology

ASN Journal Disclosure Form

As per ASN journal policy, I have disclosed any financial relationships or commitments I have held in the past 36 months as included below. I have listed my Current Employer below to indicate there is a relationship requiring disclosure. If no relationship exists, my Current Employer is not listed.

E. Saghie reports the following:
Employer: St Vincent's university hospital

I understand that the information above will be published within the journal article, if accepted, and that failure to comply and/or to accurately and completely report the potential financial conflicts of interest could lead to the following: 1) Prior to publication, article rejection, or 2) Post-publication, sanctions ranging from, but not limited to, issuing a correction, reporting the inaccurate information to the authors' institution, banning authors from submitting work to ASN journals for varying lengths of time, and/or retraction of the published work.

Name: Ellen Saghie
Manuscript ID: K360-2025-000331
Manuscript Title: Clinical Implementation of uNGAL Testing for Diagnosing Acute Kidney Injury in an Academic Tertiary Care Medical Centre
Date of Completion: May 10, 2025
Disclosure Updated Date: May 10, 2025

ASN Journal Disclosure Form

As per ASN journal policy, I have disclosed any financial relationships or commitments I have held in the past 36 months as included below. I have listed my Current Employer below to indicate there is a relationship requiring disclosure. If no relationship exists, my Current Employer is not listed.

V. Stoyanov reports the following:
Employer: St. Vincent's University Hospital, Dublin; Beacon Hospital, Dublin; and Speakers Bureau: Boehringer Ingelheim.

I understand that the information above will be published within the journal article, if accepted, and that failure to comply and/or to accurately and completely report the potential financial conflicts of interest could lead to the following: 1) Prior to publication, article rejection, or 2) Post-publication, sanctions ranging from, but not limited to, issuing a correction, reporting the inaccurate information to the authors' institution, banning authors from submitting work to ASN journals for varying lengths of time, and/or retraction of the published work.

Name: Vladimir P Stoyanov
Manuscript ID: K360-2025-000331R1
Manuscript Title: Clinical Implementation of uNGAL Testing for Diagnosing Acute Kidney Injury in an Academic Tertiary Care Medical Centre.
Date of Completion: May 11, 2025
Disclosure Updated Date: May 11, 2025

ASN
American Society of Nephrology
JASN
Journal of the American Society of Nephrology
KIDNEY360
Accessing Our World From Every Angle
CJASN
Clinical Journal of the American Society of Nephrology

ASN Journal Disclosure Form

As per ASN journal policy, I have disclosed any financial relationships or commitments I have held in the past 36 months as included below. I have listed my Current Employer below to indicate there is a relationship requiring disclosure. If no relationship exists, my Current Employer is not listed.

M. Strader reports the following:
Employer: University College Dublin; HSE

I understand that the information above will be published within the journal article, if accepted, and that failure to comply and/or to accurately and completely report the potential financial conflicts of interest could lead to the following: 1) Prior to publication, article rejection, or 2) Post-publication, sanctions ranging from, but not limited to, issuing a correction, reporting the inaccurate information to the authors' institution, banning authors from submitting work to ASN journals for varying lengths of time, and/or retraction of the published work.

Name: Michael Strader
Manuscript ID: K360-2025-000331
Manuscript Title: Clinical Implementation of uNGAL Testing for Diagnosing Acute Kidney Injury in an Academic Tertiary Care Medical Centre
Date of Completion: March 13, 2025
Disclosure Updated Date: March 12, 2025

ASN American Society of Nephrology | JASN Journal of the American Society of Nephrology | KIDNEY360 Accessing Our World From Every Angle | CJASN Clinical Journal of the American Society of Nephrology

ASN Journal Disclosure Form

As per ASN journal policy, I have disclosed any financial relationships or commitments I have held in the past 36 months as included below. I have listed my Current Employer below to indicate there is a relationship requiring disclosure. If no relationship exists, my Current Employer is not listed.

A. Tariq reports the following:
Employer: Saint Vincent's University hospital

I understand that the information above will be published within the journal article, if accepted, and that failure to comply and/or to accurately and completely report the potential financial conflicts of interest could lead to the following: 1) Prior to publication, article rejection, or 2) Post-publication, sanctions ranging from, but not limited to, issuing a correction, reporting the inaccurate information to the authors' institution, banning authors from submitting work to ASN journals for varying lengths of time, and/or retraction of the published work.

Name: Abdullah Tariq
Manuscript ID: K360-2025-000331
Manuscript Title: Clinical Implementation of uNGAL Testing for Diagnosing Acute Kidney Injury in an Academic Tertiary Care Medical Centre
Date of Completion: May 10, 2025
Disclosure Updated Date: May 10, 2025

ASN
American Society of Nephrology
JASN
Journal of the American Society of Nephrology
KIDNEY360
Accessing Our World From Every Angle
CJASN
Clinical Journal of the American Society of Nephrology

ASN Journal Disclosure Form

As per ASN journal policy, I have disclosed any financial relationships or commitments I have held in the past 36 months as included below. I have listed my Current Employer below to indicate there is a relationship requiring disclosure. If no relationship exists, my Current Employer is not listed.

P. Twomey reports the following:
Employer: University College Dublin

I understand that the information above will be published within the journal article, if accepted, and that failure to comply and/or to accurately and completely report the potential financial conflicts of interest could lead to the following: 1) Prior to publication, article rejection, or 2) Post-publication, sanctions ranging from, but not limited to, issuing a correction, reporting the inaccurate information to the authors' institution, banning authors from submitting work to ASN journals for varying lengths of time, and/or retraction of the published work.

Name: Patrick J Twomey
Manuscript ID: K360-2025-000331
Manuscript Title: Clinical Implementation of uNGAL Testing for Diagnosing Acute Kidney Injury in an Academic Tertiary Care Medical Centre
Date of Completion: March 13, 2025
Disclosure Updated Date: March 13, 2025
