# Supplementary material for: Clinical Implementation of Urinary Neutrophil Gelatinase-Associated Lipocalin Testing for Diagnosing Acute Kidney Injury in an Academic Tertiary Care Medical Centre
Source: Kidney360. 2025 Aug 13;6(12):2119–29. doi: 10.34067/KID.0000000887 (PMC12708381; doi:10.34067/KID.0000000887)
Supplement: SUPPLEMENTARY MATERIAL [file kidney360-6-2119-s002.pdf]

Supplementary Material

Supplemental Table 1 – Biochemical Data Collected from Hospital records

Supplemental Table 2 – Clinical Characteristics of Population

Supplemental Methods: Covariates for Adjusted Models

Supplemental Figure 1 – Comparison of urinary biomarker levels between adjudicated Pre-renal, Intra-renal, and Post-renal groups with and without UTI

STROBE Checklist

Supplemental Table 1. Clinical Data Collected from Hospital records

Biochemical Data Collected
as serum urea, serum creatinine, electrolytes, C-reactive protein (CRP), white blood cell count, neutrophil count, leukocyte count, serum albumin, urinary sodium, urinary Cr, urine protein:Cr ratio (PCR), albumin:Cr ratio (ACR), and uNGAL

Supplemental Table 2. – Clinical Characteristics of Population

Variable	Overall (N = 323)
Sex (Female)	121 (37.7)
Age (Mean [SD])	66.6 (16.6)
Pre-admission serum creatinine (mmol/l) (median [IQR])	107.0 [74.0, 145.0]
Serum Creatinine (mmol/L) at AKI diagnosis (median [IQR])	254.0 [179.5, 363.5]
ICU Admission (n [%])	48 (15.2)

Variable	Overall (N = 323)
Recent Surgery (n [%])	70 (21.8)
Diabetes (n [%])	64 (20.5)
Hypertension (n [%])	152 (48.4)
Cardiovascular Disease (n [%])	67 (21.3)
Stroke (n [%])	26 (8.3)
Heart Failure (n [%])	51 (16.3)
Liver Cirrhosis (n [%])	59 (19.0)
CKD (n [%])	129 (40.3)
CKD Stage (n [%])	
3	81 (62.8)
4	38 (29.5)
5	10 (7.8)
Renal Replacement therapy (n [%])	64 (19.9)
Kidney Injury Classification at uNGAL	
AKD	6 (1.9)
AKI	155 (48.0)
AKI on CKD	137 (42.4)
CKD	8 (2.5)
Undetermined	17 (5.3)
Adjudicated Pre-Renal Cause	
Heart Failure	11 (7.7)
Hepatorenal Syndrome	22 (15.4)
Hypovolemia	110 (76.9)
Active Infection	200 (62.5)
Septicemia	36 (11.1)
UTI	64 (19.8)

Variable	Overall (N = 323)
Serum WBC (Mean [Sd])	13.4 (11.7)
Serum Neutrophil Count (Mean [Sd])	10.7 (8.0)
Serum Lymphocyte Count (Mean [Sd])	1.4 (1.6)
Serum CRP (median [IQR])	56.5 [16.8, 124.0]
AKI Classification (n [%])	
Pre-renal	150 (51.4)
Intra-renal	120 (41.1)
Post-renal	22 (7.5)
Max AKI Stage (n [%])	
1	62 (20.1)
2	74 (23.9)
3	173 (56.0)

Normally distributed variables are presented as means and standard deviations (Sd), while non-normally distributed variables are presented as medians and inter quartile ranges (IQR). CKD: Chronic kidney disease; AKI: acute kidney injury; UTI: urinary tract infection; WBC: white blood cell; CRP: c-reactive protein.

Supplemental Methods: Covariates for Adjusted Models

Models were adjusted for serum Cr and serum urea levels measured on the day of AKI diagnosis, history of CKD, UTI status, and C-reactive protein (CRP) at the time of uNGAL measurement. These covariates were selected based on their routine clinical use during AKI evaluation.

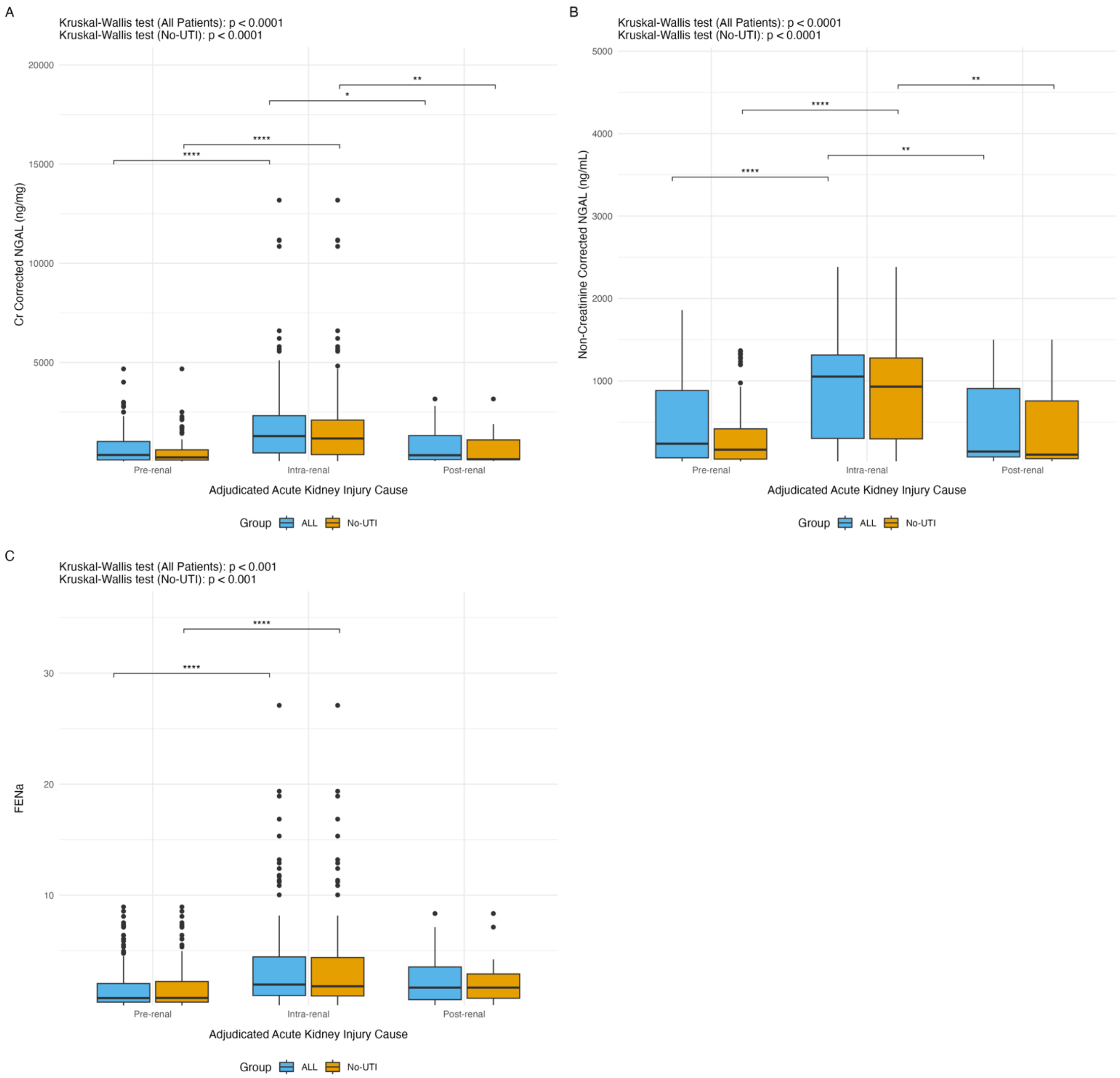
A
Kruskal-Wallis test (All Patients): p < 0.0001
Kruskal-Wallis test (No-UTI): p < 0.0001
Cr Corrected NGAL (ng/mg)
20000
15000
10000
5000
****
****
*
**
Pre-renal
Intra-renal
Post-renal
Adjudicated Acute Kidney Injury Cause
Group
ALL
No-UTI
B
Kruskal-Wallis test (All Patients): p < 0.0001
Kruskal-Wallis test (No-UTI): p < 0.0001
Non-Creatinine Corrected NGAL (ng/mL)
5000
4000
3000
2000
1000
****
**
****
**
Pre-renal
Intra-renal
Post-renal
Adjudicated Acute Kidney Injury Cause
Group
ALL
No-UTI
C
Kruskal-Wallis test (All Patients): p < 0.001
Kruskal-Wallis test (No-UTI): p < 0.001
FENa
30
20
10
****
****
Pre-renal
Intra-renal
Post-renal
Adjudicated Acute Kidney Injury Cause
Group
ALL
No-UTI

Supplemental Figure 1. Biomarker levels between adjudicated Pre-renal, Intrarenal, and Post-renal cases before and after controlling for UTI status were compared. The comparisons included: (A) Cr-corrected urinary neutrophil gelatinase-associated lipocalin (uNGAL/Cr (ng/mg)) (B) Free uNGAL levels (ng/ml) a nd (C) FENa (%) . Results of Kruskall-Wallice are presented as p values. The box-and-whisker plots depict the 25th and 75th interquartile ranges, median, and total observed ranges. Lines between groups indicate comparisons made using Dunn's post-hoc test following a significant Kruskal-Wallis test. Adjustment for the multiple comparisons was performed using the Bonferroni method. Symbols represent significance levels: * $p < 0.05$, ** $p < 0.01$, *** $p < 0.001$, **** $p < 0.0001$.

STROBE Statement

Checklist of items that should be included in reports of observational studies

Section/Topic	Item No	Recommendation	Reported on Page No
**Title and abstract**	1	(*a*) Indicate the study's design with a commonly used term in the title or the abstract	1
		(*b*) Provide in the abstract an informative and balanced summary of what was done and what was found	2,3
**Introduction**			
Background/rationale	2	Explain the scientific background and rationale for the investigation being reported	4
Objectives	3	State specific objectives, including any prespecified hypotheses	4,5
**Methods**			
Study design	4	Present key elements of study design early in the paper	4,5
Setting	5	Describe the setting, locations, and relevant dates, including periods of recruitment, exposure, follow-up, and data collection	5
Participants	6	(*a*) *Cohort study*—Give the eligibility criteria, and the sources and methods of selection of participants. Describe methods of follow-up<br>*Case-control study*—Give the eligibility criteria, and the sources and methods of case ascertainment and control selection. Give the rationale for the choice of cases and controls<br>*Cross-sectional study*—Give the eligibility criteria, and the sources and methods of selection of participants	5,6
		(*b*) *Cohort study*—For matched studies, give matching criteria and number of exposed and unexposed<br>*Case-control study*—For matched studies, give matching criteria and the number of controls per case	
Variables	7	Clearly define all outcomes, exposures, predictors, potential confounders, and effect modifiers. Give diagnostic criteria, if applicable	6,7, supplement ary Table 1
Data sources/measurement	8*	For each variable of interest, give sources of data and details of methods of assessment (measurement). Describe comparability of assessment methods if there is more than one group	6, supplement ary Table 1
Bias	9	Describe any efforts to address potential sources of bias	6-8
Study size	10	Explain how the study size was arrived at	5
Quantitative variables	11	Explain how quantitative variables were handled in the analyses. If applicable, describe which groupings were chosen and why	7,8
Statistical methods	12	(*a*) Describe all statistical methods, including those used to control for confounding	7,8
		(*b*) Describe any methods used to examine subgroups and interactions	7,8
		(*c*) Explain how missing data were addressed	7,8
		(*d*) *Cohort study*—If applicable, explain how loss to follow-up was addressed<br>*Case-control study*—If applicable, explain how matching of cases and controls was addressed<br>*Cross-sectional study*—If applicable, describe analytical methods taking account of sampling strategy	
		(*e*) Describe any sensitivity analyses	7,8

Section/Topic	Item No	Recommendation	Reported on Page No
**Results**			
Participants	13*	(a) Report numbers of individuals at each stage of study—eg numbers potentially eligible, examined for eligibility, confirmed eligible, included in the study, completing follow-up, and analysed	9
		(b) Give reasons for non-participation at each stage	9
		(c) Consider use of a flow diagram	Fig 1
Descriptive data	14*	(a) Give characteristics of study participants (eg demographic, clinical, social) and information on exposures and potential confounders	9,10
		(b) Indicate number of participants with missing data for each variable of interest	9,10
		(c) *Cohort study*—Summarise follow-up time (eg, average and total amount)	9,10
Outcome data	15*	*Cohort study*—Report numbers of outcome events or summary measures over time	9,10
		Case-control study—Report numbers in each exposure category, or summary measures of exposure	
		Cross-sectional study—Report numbers of outcome events or summary measures	
Main results	16	(*a*) Give unadjusted estimates and, if applicable, confounder-adjusted estimates and their precision (eg, 95% confidence interval). Make clear which confounders were adjusted for and why they were included	9-13
		(*b*) Report category boundaries when continuous variables were categorized	
		(*c*) If relevant, consider translating estimates of relative risk into absolute risk for a meaningful time period	
Other analyses	17	Report other analyses done—eg analyses of subgroups and interactions, and sensitivity analyses	10-13
**Discussion**			
Key results	18	Summarise key results with reference to study objectives	13-15
Limitations	19	Discuss limitations of the study, taking into account sources of potential bias or imprecision. Discuss both direction and magnitude of any potential bias	15,16
Interpretation	20	Give a cautious overall interpretation of results considering objectives, limitations, multiplicity of analyses, results from similar studies, and other relevant evidence	13-15
Generalisability	21	Discuss the generalisability (external validity) of the study results	16,17
**Other Information**			
Funding	22	Give the source of funding and the role of the funders for the present study and, if applicable, for the original study on which the present article is based	18

**Give information separately for cases and controls in case-control studies and, if applicable, for exposed and unexposed groups in cohort and cross-sectional studies.*

Note: An Explanation and Elaboration article discusses each checklist item and gives methodological background and published examples of transparent reporting. The STROBE checklist is best used in conjunction with this article (freely available on the Web sites of PLoS Medicine at http://www.plosmedicine.org/, Annals of Internal Medicine at http://www.annals.org/, and Epidemiology at http://www.epidem.com/). Information on the STROBE Initiative is available at www.strobe-statement.org.
